# Comparison of the efficacy of subthreshold micropulse laser versus intravitreal anti-VEGF therapy for chronic central serous chorioretinopathy

**DOI:** 10.3389/fmed.2026.1832055

**Published:** 2026-04-30

**Authors:** Jinyu Li, Tiepei Zhu, Yanan Zhu, Yao Wang, Haixiu Wu, Zhiqing Chen

**Affiliations:** Eye Center, The Second Affiliated Hospital, School of Medicine, Zhejiang University, Zhejiang Provincial Key Laboratory of Ophthalmology, Zhejiang Provincial Clinical Research Center for Eye Diseases, Zhejiang Provincial Engineering Institute on Eye Diseases, Hangzhou, Zhejiang, China

**Keywords:** chronic central serous chorioretinopathy (cCSCR), intravitreal anti-VEGF injection (IVA), subfoveal choroidal thickness (SFCT), subretinal fluid (SRF), subthreshold micropulse laser (SML)

## Abstract

**Objective:**

To compare the therapeutic ability between 577-nm subthreshold micropulse laser (SML) and intravitreal anti-VEGF injection (IVA) in the treatment of chronic central serous chorioretinopathy (cCSCR).

**Methods:**

The medical records of 50 cCSCR patients (50 eyes) who underwent SML (30 eyes) or IVA injection (20 eyes) were reviewed in this retrospective cohort study. Changes in best-corrected visual acuity (BCVA), central retinal thickness (CRT), subretinal fluid (SRF), and subfoveal choroidal thickness (SFCT) measured by optical coherence tomography (OCT) at baseline, 1, 3, and 6 months post-treatment were extracted and compared between the two groups.

**Results:**

Baseline demographic and clinical characteristics, including age, sex, leakage pattern on fluorescein angiography (FA), BCVA, CRT, SRF, and SFCT, were comparable between the two groups (all *p* > 0.05). The mean ± SD of SML sessions and IVA injection were 1.67 ± 0.80 and 1.75 ± 0.97, respectively (*p* > 0.05). In comparison to the baseline values, BCVA showed statistically significant improvement at 6 months after treatment in both groups (*p* < 0.05), while the CRT, SRF and SFCT values prominently decreased at the final evaluation (all *p* < 0.05). No statistically significant difference was revealed in the BCVA and CRT data of all time points between the two groups (all *p* > 0.05). At baseline, 1 month, and the 3 months, there was no statistically significant difference for the SRF and SFCT values between groups (*p* > 0.05). However, at the 6th month, the SML group demonstrated significantly lower SRF, whereas the IVA group showed significantly lower SFCT (both *p* < 0.05).

**Conclusion:**

Both SML and anti-VEGF has been confirmed effective in improving visual acuity and achieving anatomical recovery in patients with cCSCR. At 6 months, IVA was associated with greater reduction in SFCT, whereas SML achieved superior SRF resolution. These findings support individualized treatment selection based on disease characteristics and underlying pathophysiology.

## Introduction

Central serous chorioretinopathy is a common macular disease characterized by idiopathic serous detachment of the neurosensory retina on account of functional impairment of the retinal pigment epithelium (RPE). It primarily affects young and middle-aged males, with a morbidity of 9.9 per 100,000 in men and 1.7 per 100,000 in women (1). While most cases can spontaneously recover within 3–6 months, some patients experience recurrent and chronic disease, which can lead to irreversible visual loss in the working population. Consequently, it is increasingly recognized as a significant public health problem (2).

Many treatment strategies, including photodynamic therapy (PDT), laser therapy and pharmacology like corticosteroid receptor antagonist, have been used for CSCR (3). The PLACE trial and other studies confirmed the positive effects achieved by PDT with verteporfin in CSCR (4, 5). However, PDT can lead to potential adverse events, such as RPE atrophy and choroidal capillary hypoperfusion (6). Half-dose PDT is universally recognized as the first-line treatment for chronic CSC according to the latest consensus guidelines (7). A randomized, double-blind trial showed that eplerenone was not superior to placebo in improving BCVA in chronic CSCR (8). Conventional Laser photocoagulation (CLP) is used for leakage area away from the fovea, but it may cause retinal thermal burns, dark spots for patients, CNV, and new leakage sites (9). The goal of CLP is to achieve light intensity combustion in the leakage area. However, due to the retinal burn effect, this treatment can also cause side effects, such as visual field defects, preretinal membrane and CNV (10). In order to reduce retinal damage, other wavelengths and techniques have been introduced, such as subthreshold micropulse laser (SML) (11). SML has been reported to be effective in CSCR as it can targeted promote the retinal pigment epithelial cells’ (RPE) migration and proliferation, which induces RPE regeneration, with no effect on neurosensory retina (12). Anti-VEGF has also been explored as a therapy for CSCR in previous studies (13, 14), and a meta-analysis has partially indicated that it might be a viable choice for chronic CSCR, although the use of anti-VEGF in CSCR remains off-label (15).

The study aimed to analyze the clinical effectiveness and anatomical changes of SML and IVA in the treatment of cCSCR using multimodal imaging examination.

## Materials and methods

### Study design, participants, and ethics approval

This was a single-center retrospective study comparing the efficacy and safety of SML versus IVA in the treatment of cCSCR. The study protocol was approved by the Ethics Committee of the Second Affiliated Hospital, School of Medicine, Zhejiang University. The study adhered to the tenets of the Declaration of Helsinki. Given the retrospective design and the use of de-identified clinical and imaging data without influencing patient management, the Institutional Review Board waived the requirement for written informed consent for study participation. Written informed consent for therapeutic procedures had been obtained from all patients as part of routine clinical care.

Fifty patients with cCSCR from January 2018 to December 2022 were reviewed. They were retrospectively assigned to the SML or IVA group according to the treatment they had received. All patients underwent relevant clinical examinations of eye, containing best corrected visual acuity (BCVA), SD-OCT (Spectralis OCT, Heidelberg Engineering GmbH, Germany), FA and indocyanine green angiography (ICGA) (Spectralis HRA, Heidelberg Engineering GmbH, Germany). OCTA (OptovueRTVue XR, Optovue, Fremont, CA, United States) was conducted only in patients with suspected CNV detected on FA or ICGA. The diagnosis of cCSCR was defined as the presence of subretinal fluid persisting for more than 6 months.

The inclusion criteria were as follows: (1) Patients aged 18–80 years; (2) Patients with the foveal neurosensory retinal detachment on OCT; (3) Patients who received either micropulse laser therapy or anti-VEGF injection as the primary treatment for cCSCR; (4) Presence of at least 6 months of follow-up data. The exclusion criteria included: (1) Pregnancy; (2) Patients with uncontrolled ocular disease, covering age-related macular degeneration, polypoid choroidal vasculopathy, pathological myopia; (3) Patients with systemic diseases such as systemic lupus erythematosus; (4) Bullous retinal detachment; (5) A history of receiving conventional laser and PDT before recruitment; (6) Receipt of intravitreal anti-VEGF injection, within 3 months prior to the index treatment for cCSCR, only in the study eye, for any retinal indication including cCSCR.

### Subthreshold micropulse laser

Pupil dilation was achieved using tropicamide eye drops. After local corneal anesthesia with Elcaine eye drops, a contact lens with carbomer as the contact gel was placed over the eye. Following to a previously reported protocol (16, 17), SML was performed by a single ophthalmologist (CZQ) using a 577 nm micropulse laser mode (Quantel Medical). The spot diameter was set to 160 μm, the duration was 0.2 s, and the duty cycle was 5% (0.1 ms on, 0.9 ms off) to cover the region of detachment of the neurosensory retina detected by optical coherence tomography (OCT). Power titration was executed in a single point micropulse pattern, starting at 600 mW. Then, the energy was elevated to produce light white spots in the peripheral retinal region near the vascular arch. In the macula, half of the power levels in the micropulse pattern were utilized by confluence laser points. The quantity of spots depends on the extent of macular edema and the spread of fluorescent leakage areas. SML treatment would be repeated if neuroepithelial detachment persisted or recurred, but no earlier than one month after the previous treatment.

### Intravitreal anti-VEGF injection

All injections were performed under operating room conditions. After disinfecting the eyes with 5% povidone iodine, and under local anesthesia, the eyelids were stabilized with a blepharostat and anti-VEGF drugs were injected. Intravitreal injections of aflibercept (2 mg/0.05 mL) were administered. Patients were instructed to use antibiotic eye drops for 3 days before and after the injection.

### Efficacy evaluation

Clinical examination data at approximately 1, 3, and 6 months after treatment routinely collected and analyzed by two independent retinal specialists (WY and LJY), including BCVA, which were converted to logarithms of the minimum Resolution Angle (logMAR) scale, central retinal thickness (CRT), subretinal fluid (SRF), subfoveal choroidal thickness (SFCT) from OCT. CRT was assessed using manual measurements of the distance between the inner boundary membrane of the OCT fovea and the RPE. SFCT measurements were collected under enhanced depth imaging (EDI) mode.

### Statistical analysis

Statistical analyses were performed using R software (version 3.3.3; The R Foundation for Statistical Computing, Vienna, Austria), Python (version 3.11.0; Python Software Foundation, Wilmington, DE, United States), and GraphPad Prism (version 9.0; GraphPad Software, San Diego, CA, United States). Continuous variables were expressed as mean ± standard deviation (SD), and categorical variables were presented as frequencies and percentages. Longitudinal changes in best-corrected visual acuity (BCVA), central retinal thickness (CRT), subretinal fluid (SRF), and subfoveal choroidal thickness (SFCT) across routine follow-up records were analyzed using repeated-measures analysis of variance (ANOVA). Intergroup differences over time were assessed using repeated-measures ANOVA with treatment group as the between-subject factor and follow-up time as the within-subject factor. Comparisons between the two treatment groups were performed using the independent-samples *t*-test for normally distributed data and the Mann–Whitney U test for non-normally distributed data. A *p* < 0.05 was considered statistically significant.

## Results

### Baseline demographic and clinical characteristics

Fifty patients were included in the study, comprising 30 eyes in the SML group and 20 eyes in the IVA group. The mean age of the patients was 50.6 ± 9.4 years. There were no significant intergroup differences at baseline in terms of gender, age, BCVA (logMAR), CRT, or SFCT (all *p* > 0.05). Detailed demographic and clinical characteristics are summarized in Table 1. The mean number of SML and anti-VEGF treatments was 1.67 ± 0.80 and 1.75 ± 0.97, respectively. There were no significant differences in the number of administered IVA injections and SML sessions between groups (Table 2).

**TABLE 1:** Baseline demographics and clinical characteristics of SML and IVA groups.

Characteristic	SML (*N* = 30)	IVA (*N* = 20)	*p*-value
Demographics			
Number of eyes	30	20	N/A
Gender (male,%)	73.33%	70.00%	0.140
Age (years)	49.23 ± 8.90	52.70 ± 9.99	0.205
Eye (Right, %)	16.53%	12.60%	N/A
Characteristics of the FA (type of leakage)			
Focal	56.67%	60%	N/A
Diffuse	43.33%	40%	
Characteristics of BCVA and OCT			
BCVA (logMAR), (mean ± SD)	0.37 ± 0.17	0.35 ± 0.12	0.539
CRT (mean ± SD), μm	425.27 ± 108.51	379.30 ± 115.83	0.168
SRF (mean ± SD), μm	258.00 ± 101.21	224.60 ± 102.19	0.270
SFCT (mean ± SD), μm	429.83 ± 43.93	445.65 ± 31.83	0.181

SD, standard deviation; SML, Subthreshold Micropulse Laser; IVA, Intravitreal injection of anti-vascular endothelial growth factor; CSCR, central serous chorioretinopathy; BCVA, best-corrected visual acuity; logMAR, logarithm of the minimum angle of resolution; CRT, Central retinal thickness; SRF, subretinal fluid; SFCT, Subfoveal choroidal thickness.

**TABLE 2:** Number of treatment sessions in the SML and IVA groups.

Group	Range	Mean ± SD	*p-*value
SML group	1–4	1.67 ± 0.80	0.75
IVA group	1–4	1.75 ± 0.97	

### Adverse Events

No patients experienced intraocular inflammation, hemorrhage, or other treatment-related adverse events following SML or intravitreal injection.

### Treatment Effects on BCVA, CRT, SRF, and SFCT

No statistically difference was observed between the two groups in SRF or SFCT values at baseline, 1 month, or 3 months after treatment (*p* > 0.05). However, at the 6-month follow-up visit, SRF was statistically significantly lower in the SML group (53.600 ± 52.081 vs. 96.450 ± 75.923 μm, P = 0.025), whereas SFCT was statistically significantly lower in the IVA group (353.400 ± 26.830 vs. 389.500 ± 50.470 μm, *P* = 0.006). No statistically significant intergroup differences were detected in BCVA or CRT at any follow-up time points (all p > 0.05). Detailed intergroup comparison of BCVA, CRT, SRF and SFCT data are listed in Table 3.

**TABLE 3:** Comparison of BCVA, CRT, SRF height, and SFCT between the SML and IVA groups during the 6-month follow-up.

Parameter	Time	SML group (mean ± SD)	IVA group (mean ± SD)	*p*-value
BCVA (logMAR)	Baseline	0.374 ± 0.169	0.346 ± 0.122	0.539
	1 Month	0.299 ± 0.164	0.279 ± 0.117	0.651
	3 Months	0.238 ± 0.155	0.211 ± 0.096	0.512
	6 Months	0.187 ± 0.139	0.176 ± 0.163	0.815
CRT (μm)	Baseline	425.267 ± 108.509	379.300 ± 115.831	0.168
	1 Month	317.333 ± 68.658	298.100 ± 65.098	0.344
	3 Months	265.800 ± 61.253	278.150 ± 66.187	0.508
	6 Months	225.700 ± 42.198	255.950 ± 73.747	0.077
SRF (μm)	Baseline	258.000 ± 101.209	224.600 ± 102.190	0.270
	1 Month	143.367 ± 63.955	141.750 ± 64.890	0.932
	3 Months	87.100 ± 66.347	121.200 ± 65.708	0.086
	6 Months	53.600 ± 52.081	96.450 ± 75.923	0.025*
SFCT (μm)	Baseline	429.833 ± 43.933	445.650 ± 31.825	0.181
	1 Month	413.767 ± 40.563	402.450 ± 29.360	0.298
	3 Months	404.166 ± 43.240	385.850 ± 31.240	0.116
	6 Months	389.500 ± 50.470	353.400 ± 26.830	0.006*

Bold values indicate statistically significant between-group differences at 6 months (*p < 0.05).

**FIGURE 1:**
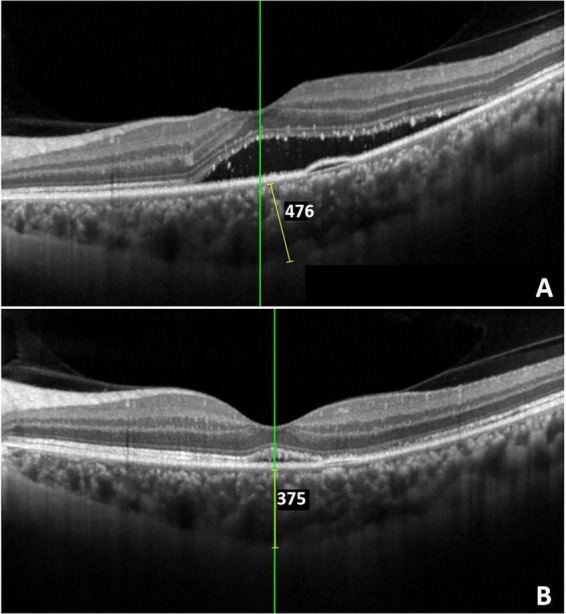
Evaluation of subfoveal choroidal thickness (SFCT) using enhanced depth imaging (EDI). Representative images demonstrating SFCT measurements before **(A)** and after **(B)** SML treatment under EDI mode. Following SML, SRF was nearly completely resolved, and SFCT decreased from 476 to 375 μm.

Compared to the baseline, considerable improvement for BCVA was observed at the 3, 6 months in two groups (*p* < 0.05). CRT and SRF values significantly decreased at 1, 3, 6 months of both groups (all *p* < 0.05). In the IVA group, SFCT substantially decreased at all-time points of follow-up compared with baseline (p < 0.05). In contrast, a significant reduction in SFCT was observed only at the 6-month follow-up visit in the SML group (Table 4). Representative imaging from a patient treated with SML demonstrated near-complete resolution of SRF, with SFCT decreasing from 476 to 375 μm (Figure 1).

**TABLE 4:** Within-group comparison of BCVA, CRT, SRF, and SFCT changes from baseline.

Parameter	1 month	3 months	6 months
SML group			
BCVA	0.263	0.007*	< 0.001*
CRT	< 0.001*	<0.001*	< 0.001*
SRF	< 0.001*	<0.001*	< 0.001*
SFCT	0.522	0.133	0.005*
IVA group			
BCVA	0.362	0.008*	< 0.001*
CRT	0.018*	0.002*	< 0.001*
SRF	0.009*	< 0.001*	<0.001*
SFCT	< 0.001*	<0.001*	< 0.001*

*p < 0.05.

## Discussion

The pathogenesis of CSCR remains incompletely understood, and its optimal management continues to be debated (18). Current therapeutic options, including PDT, SML, and anti-VEGF therapy, demonstrate variable treatment responses among patients (19). Recent meta-analyses and the PLACE Trial have shown that these interventions provide superior outcomes compared with observation alone, with half-dose or half-fluence PDT being the most effective (4, 20, 21). Nevertheless, both SML and anti-VEGF therapy have been reported to achieve favorable outcomes in selected CSCR cases (22, 23). In China, the limited availability of verteporfin has further promoted the clinical application of SML and anti-VEGF therapy in recent years (24, 25).

A recent retrospective study comparing SML with anti-VEGF for cCSCR demonstrated comparable efficacy for both interventions within a 1-month follow-up period (25). Our study expands upon these findings by evaluating longer-term outcomes. Our results demonstrated significant improvements in BCVA and reductions in CRT, SRF, and SFCT at 3 and 6 months following treatment in both groups. These findings indicate that both SML and anti-VEGF therapy contribute to structural and functional recovery in patients with chronic CSCR during long-term follow-up. No significant intergroup differences in SRF or SFCT were observed at 1 or 3 months after treatment. However, at the 6-month follow-up, the SML group demonstrated greater SRF resolution, whereas the IVA group showed a more pronounced reduction in SFCT. These observations suggest that the two treatments may exert therapeutic effects through different biological mechanisms (26).

**FIGURE 2:**
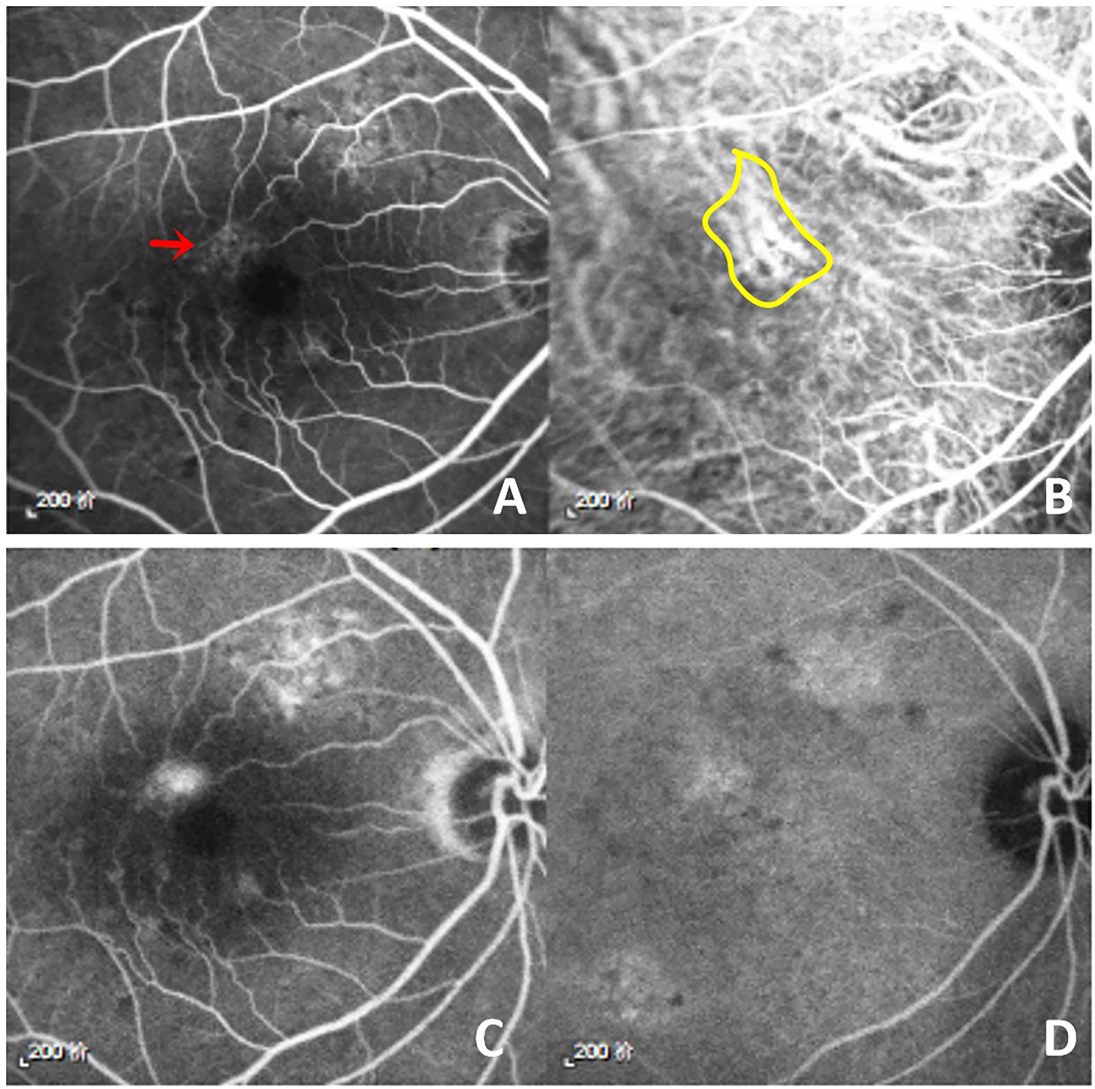
Choroidal vascular hyperpermeability in a patient with CSCR. Multimodal imaging showing choroidal vascular hyperpermeability and leakage on fluorescein angiography (FA) and indocyanine green angiography (ICGA). **(A)** Early-phase FA. **(B)** Early-phase ICGA. Hyperfluorescence indicates active leakage (red arrow) on FA and dilated choroidal vessels (yellow circle) on ICGA. **(C)** Late-phase FA. **(D)** Late-phase ICGA.

The pachychoroid disease spectrum has been increasingly recognized as a core pathogenic framework for CSCR (27). Choroidal hyperpermeability and retinal pigment epithelium (RPE) dysfunction are considered key contributors to disease development (26, 28, 29). Dysregulation of choroidal vasculature is regarded as a central pathological feature of CSCR and is frequently demonstrated by ICGA findings, including dilated and hyperpermeable choroidal vessels (30). In our study, increased choroidal vascular permeability was observed in patients with ICGA evaluation at baseline (Figure 2). Previous studies have also suggested that venous congestion within the vortex vein system may contribute to CSCR pathogenesis (31). Structural alterations involving the choroid, sclera, and RPE might be related to venous congestion of choroidal outflow, resulting in dilation of Haller vessels, thinning of the choriocapillaris and Sattler’s layer, and abnormalities of subsequent RPE dysfunction leading to fluid leakage (32).

**FIGURE 3:**
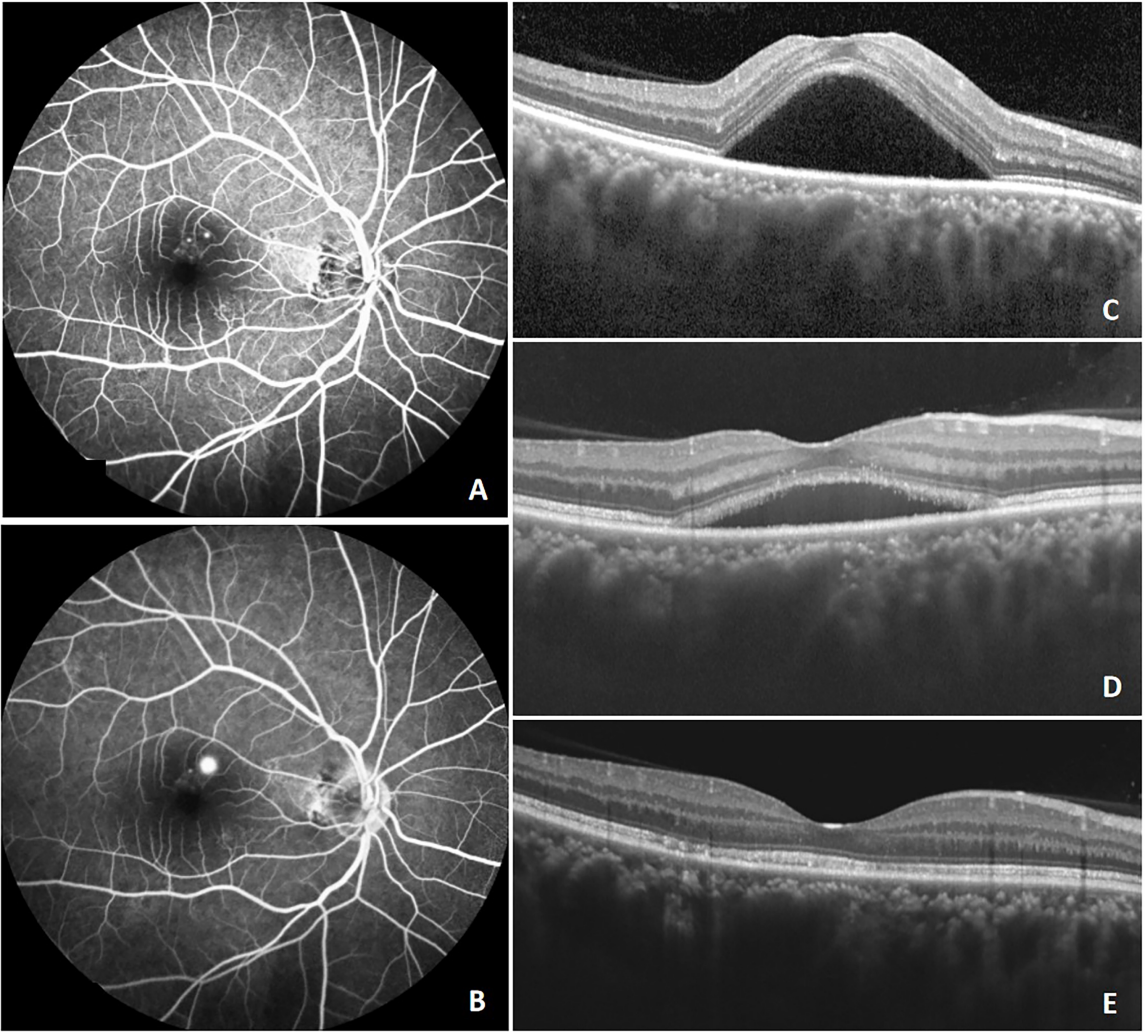
Multimodal imaging of a patient with favorable response to SML. A 36-year-old man diagnosed with chronic CSCR underwent three sessions of SML treatment. At baseline **(A–C)**, focal hyperfluorescence was observed on FA (**A:** early phase; **B:** late phase), and SRF was detected on OCT **(C)**. At 1 month after treatment, SRF was partially resolved **(D)**. At 6 months, SRF was completely absorbed **(E)**.

**FIGURE 4:**
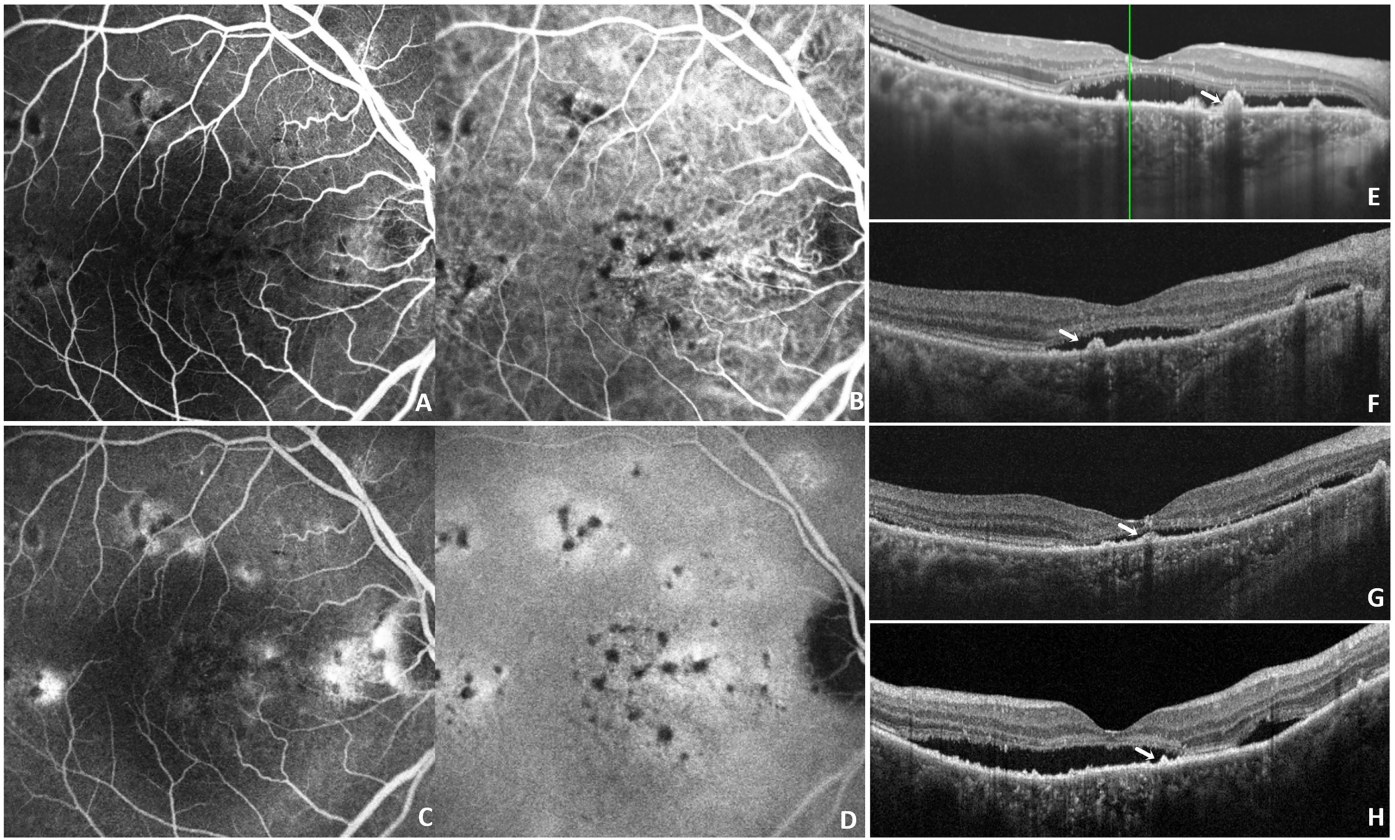
Multimodal imaging of a patient with suboptimal response to SML. A 55-year-old man with chronic CSCR underwent three sessions of SML treatment. Early-phase FA **(A)** and late-phase FA **(C)** demonstrated multiple hyperfluorescent leakage spots. Early-phase ICGA **(B)** revealed choroidal vascular hyperpermeability, and hypofluorescent spots were observed in both early **(B)** and late **(D)** phases of ICGA. At baseline **(A–E)**, OCT showed neurosensory retinal detachment in the macular region and multiple irregular pigment epithelial detachments (PEDs) (white arrow) **(E)**. After three SML sessions, SRF was gradually reduced but persisted, multiple PEDs persisted **(F,G)**. At the final visit, recurrence of SRF in the fovea was observed on OCT **(H)**.

Selective targeting of RPE cells represents a fundamental therapeutic feature of SML (33). It delivers sublethal energy to the cell without directly affecting adjacent cells such as photoreceptor cells (34). Sublethal thermal stimulation can induce heat shock protein expression, which plays a role in inhibiting apoptosis and modulating inflammatory responses (35). Therefore, SML may facilitate restoration of RPE barrier function and impedes fluid from passing through the choroid (36). SML could improve both anatomical outcomes and visual acuity in acute and chronic CSCR according to previous studies (37, 38). Additionally, reductions in choroidal thickness following SML have been reported, possibly reflecting improved vascular integrity and reduced interstitial fluid accumulation (39, 40). Consistent with these observations, our study demonstrated that SML contributed to SRF resolution and reductions in choroidal thickness in most cases (Figure 3). Favorable treatment outcomes were more frequently observed in patients presenting with lower SRF height and smaller pigment epithelial detachment (PED). Conversely, patients with marked baseline SFCT, extensive RPE dysfunction, inner choroidal attenuation, irregular PED morphology, or diffuse choroidal hyperpermeability tended to demonstrate suboptimal responses to SML (Figure 4). These findings suggest that the therapeutic efficacy of SML may depend on preserved RPE functional capacity.

**FIGURE 5:**
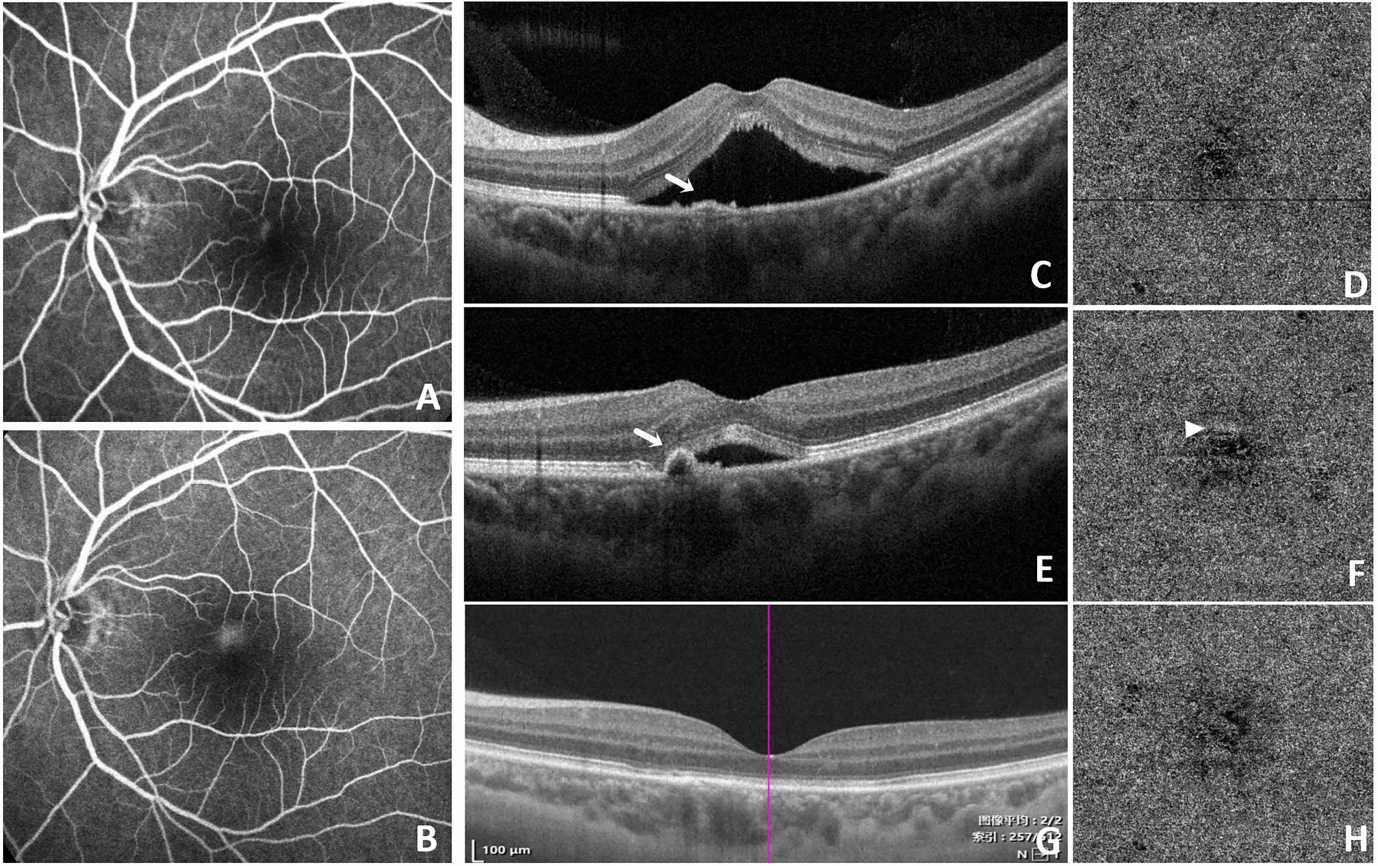
Multimodal imaging of a patient with favorable response to IVA. A 42-year-old man with chronic CSCR received IVA treatment. Early-phase FA **(A)** and late-phase FA **(B)** showed focal hyperfluorescent leakage spots. According to OCT **(C,E,G)** and choriocapillaris layer of OCTA **(D,F,H)** images, SRF resolved markedly after two IVA injections. Irregular PEDs was showed in OCT imaging (white arrow) **(C,E)**. Heterogeneous hyperreflectivity in the choriocapillaris layer (white triangle) may represent suspected CNV developing during disease progressionn **(F)**.

**FIGURE 6:**
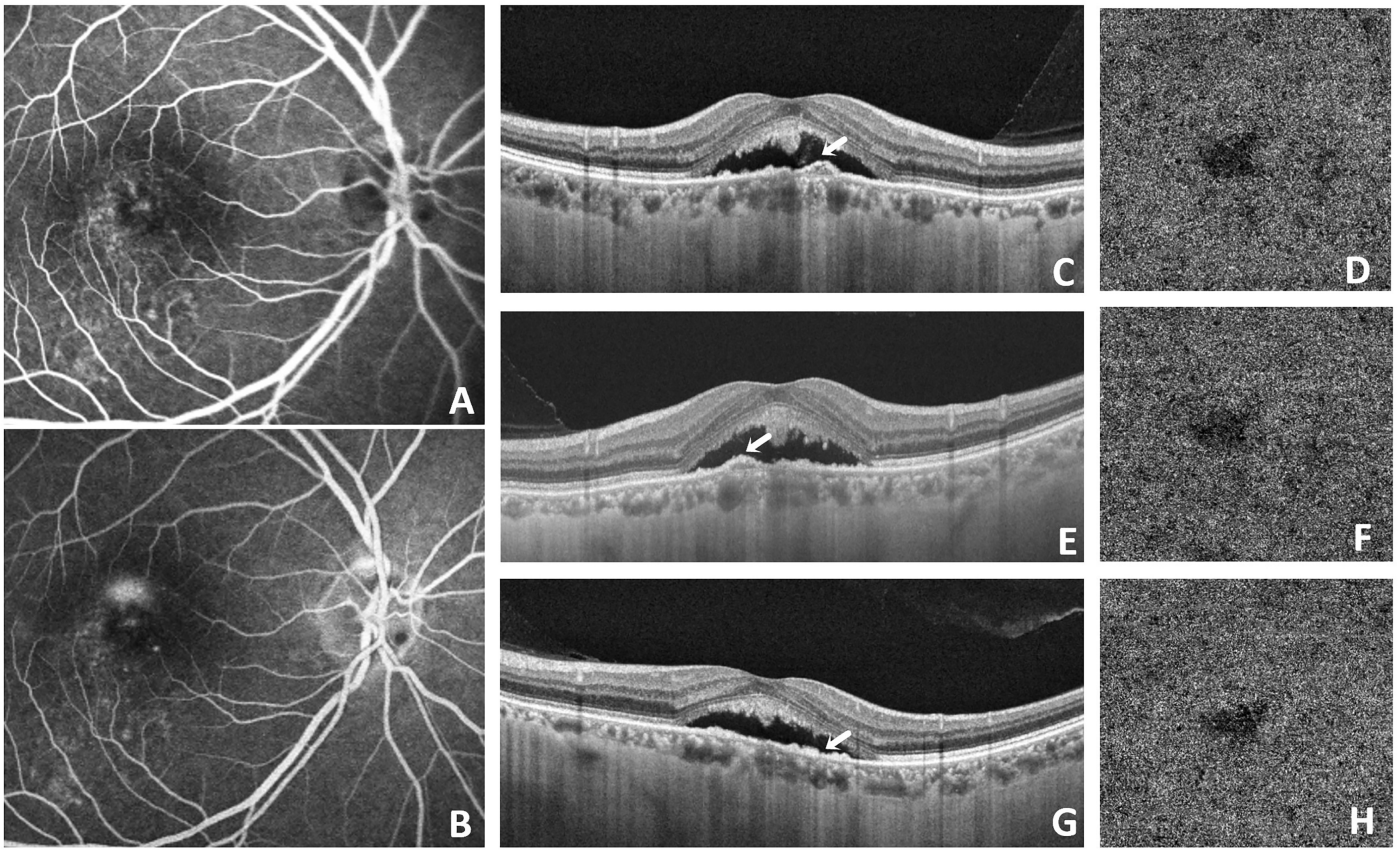
Multimodal imaging of a patient with suboptimal response to IVA. A 63-year-old woman with a 3-year history of CSCR was treated with IVA. At baseline, focal hyperfluorescence was observed on FA (**A:** early phase; **B:** late phase). Based on the findings on OCT **(C,E,G)** and choriocapillaris layer of OCTA **(D,F,H)**, this patient demonstrated a suboptimal response to IVA treatment. OCT revealed neurosensory retinal detachment in the macular region with PEDs (white arrow) **(C,E,G)**. After three IVA injections, SRF still exists **(G)**.

CSCR is also considered an early manifestation within the pachychoroid disease spectrum (41). Progressive choriocapillaris ischemia may promote the development of CNV at sites of pachyvessels, a condition termed pachychoroid neovasculopathy (PNV) (42). Anti-VEGFs has been shown to effectively improve visual acuity and retinal thickness outcomes in patients with PNV (43, 44). However, its application in CSCR without overt CNV remains controversial (45, 46). Previous studies have reported elevated VEGF concentrations in both serum and aqueous humor of patients with CSCR, particularly in chronic cases presenting with flat irregular pigment epithelial detachment (FIPED) associated with CNV (47, 48). Retrospective studies have suggested that non-homogenous hyperreflectivity in the choriocapillaris layer on OCTA may represent putative CNV in CSCR (14, 49). Similar imaging characteristics suggestive of subclinical neovascularization were observed in patients in our cohort (Figure 5). In contrast, patients with a longer disease duration or absence of CNV-related biomarkers at baseline tended to show less satisfactory responses (Figure 6). These observations suggest that anti-VEGF therapy may primarily target putative neovascular and choroidal vascular abnormalities within the pachychoroid disease process.

In this study, greater SRF resolution was observed in the SML group, whereas a larger decrease in SFCT was observed in the IVA group during the 6-month follow-up. These findings may be related to the distinct mechanisms of action of the two treatments. SML primarily targets RPE cells, whereas IVA mainly acts on the choroidal vasculature. Previous studies have suggested that IVA can effectively reduce SFCT in pachychoroid-related diseases (50). In our cohort, patients treated with SML appeared more likely to be in an earlier stage of the disease, when RPE function may still be relatively preserved. Stimulation of the RPE by SML might facilitate subretinal fluid resorption, resulting in more pronounced SRF resolution. In contrast, when choroidal vascular abnormalities are more prominent or when putative CNV is present, IVA may exert greater therapeutic effects, which may be reflected by a greater reduction in SFCT. Comprehensive baseline evaluation using multimodal imaging, including OCT, OCTA, FA, and ICGA, is essential for guiding individualized treatment strategies. In particular, ICGA and high-resolution OCTA may improve detection of suspected CNV and facilitate more accurate disease phenotyping (51). The present study also highlights the importance of disease stage in determining treatment selection and therapeutic outcomes.

This study has several limitations. First, the retrospective design may introduce selection bias and confounding factors, limiting causal inference. The baseline clinical characteristics suggests physician-driven treatment selection. Second, the absence of a randomized control group precluded direct comparisons with untreated controls or other established therapeutic strategies, such as PDT, and limited the strength of treatment efficacy evaluation. Finally, the relatively small sample size may limit the generalizability of the results. Future prospective randomized controlled studies with standardized multimodal imaging are warranted.

## Conclusion

In conclusion, both SML and IVA demonstrated favorable visual and anatomical outcomes in patients with cCSCR. When verteporfin-based photodynamic therapy is unavailable, these modalities may serve as viable alternative treatment options. SML was associated with greater SRF resolution at 6 months, whereas IVA produced a more pronounced reduction in SFCT. These findings suggest that SML and anti-VEGF may have different roles in cCSCR management, and that treatment selection based on imaging phenotype should be further evaluated in prospective studies. Our findings support an individualized treatment approach guided by disease stage and underlying pachychoroid-driven pathophysiology.

## Data Availability

The original contributions presented in the study are included in the article/supplementary material, further inquiries can be directed to the corresponding author.
